# A Curious Case of Intermittent Left Bundle Branch Block Associated with Cough

**DOI:** 10.7759/cureus.3520

**Published:** 2018-10-30

**Authors:** Hunaina Shahab, Osman Faheem, Kumail Khandwala, Aamir H Khan

**Affiliations:** 1 Cardiology, Aga Khan University Hospital, Karachi, PAK; 2 Radiology, Aga Khan University Hospital, Karachi, PAK

**Keywords:** left bundle-branch block (lbbb), cough, electrocardiogram (ecg), voluntary control of lbbb

## Abstract

A handful of cases of voluntary control of left bundle branch block (LBBB) have been described in the literature. We report the case of a middle-aged man who was found to have LBBB on baseline electrocardiogram (ECG) which disappeared on coughing and then reappeared with the same maneuver. Subsequent myocardial perfusion scan showed reduced count in the anteroseptal region likely attributed to LBBB. It is possible that the intermittent conduction changes may be due to the alteration in the vagal tone associated with cough as reflected in the change in the PR interval on the ECG.

## Introduction

Eppinger and Rothberger in the 1900s showed that the section of the left bundle branch produced a characteristic change in the electrocardiogram (ECG) which was later known to be the left bundle branch block (LBBB) [1]. The prevalence of LBBB is 1.1% [2]. In 1979, the Framingham study prospectively showed that the people who developed LBBB were mostly those with prior hypertension, coronary artery disease, or cardiac dilatation and within a decade, 50% of these people died of cardiovascular causes [3]. The mortality risk associated with LBBB prompts further investigation ranging from noninvasive to invasive in selected cases [4-5]. There is very little literature on the voluntary control of LBBB; however, we describe the case of a middle-aged man with a history of atypical chest pain and cough who presented with LBBB which reverted to narrow complex on coughing and back to LBBB on the same maneuver.

## Case presentation

A 57-year-old man, who was diabetic, hypertensive and had a family history of ischemic heart disease, presented to the cardiology clinic at the Aga Khan University Hospital, Karachi, Pakistan. He had a history of retrosternal chest burning after meals and occasional chest heaviness at rest with no relationship to exertion. He had been complaining of a dry cough for the past four to five days. He was functional class I. On clinical examination, he had a blood pressure of 138/84 mmHg and a heart rate of 76 beats per minute, with an oxygen saturation of 98% on room air. Cardiac auscultation revealed S1 and S2 with no added heart sounds. Chest examination revealed normal vesicular breathing. Rest of the systemic examination was also within normal limits.

**Figure 1:**
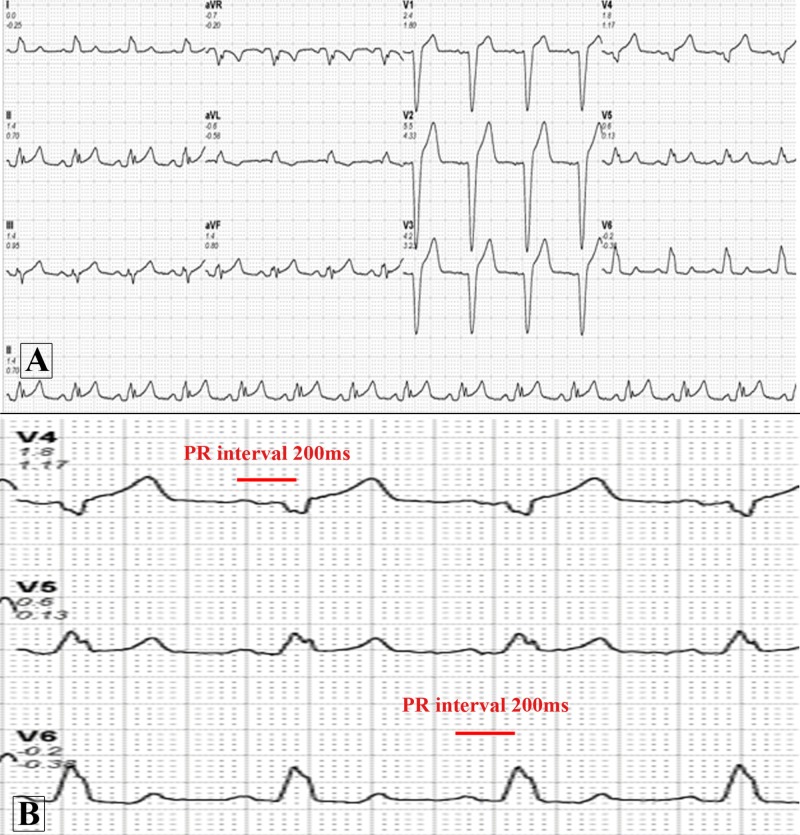
Baseline electrocardiogram of the patient. (A) Normal sinus rhythm at 83 beats per minute with a normal axis and a left bundle branch block; (B) Zoomed image of leads V4 to V6 showing the PR interval of 200 milliseconds.

Baseline ECG showed normal sinus rhythm with LBBB. Blood workup included complete blood count, creatinine, and electrolytes which were within normal ranges. Two sets of troponin I conducted from the clinic were negative. He was then referred for a myocardial perfusion scan with dipyradimole for ischemia assessment. Baseline ECG is shown in Figure 1A-1B. As per our institutional protocol, same day rest single photon emission computed tomography (SPECT) imaging was obtained with 760 MBq of intravenous (IV) Tc-99m tetrofosmin.

**Figure 2:**
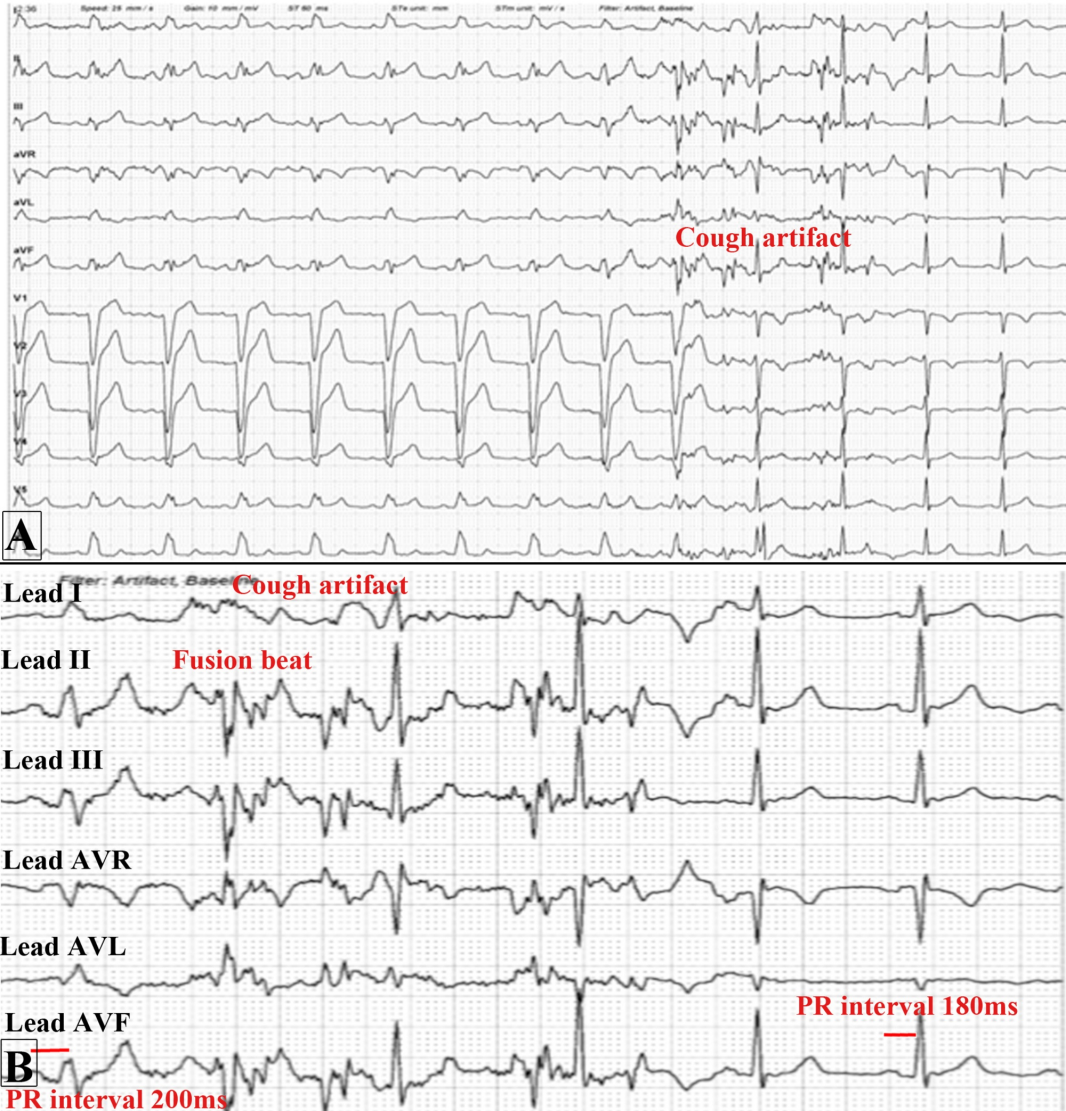
Electrocardiogram (ECG) at the start of the stress part of the myocardial perfusion scan before injecting IV dipyradimole. (A) It shows the conversion of the baseline left bundle branch block to narrow QRS complexes after a bout of cough as depicted by the artifact in the ECG; (B) Zoomed image of the ECG which shows the limb leads I, II, III, augmented vector right (AVR), augmented vector left (AVL), and augmented vector foot (AVF) showing the subtle decrease in PR interval from 200 milliseconds before the cough with wide QRS complexes to 180 milliseconds afterward with narrow QRS complexes.

At the start of the stress part of the test, the patient had a bout of dry cough. The ECG monitor documented a conversion of the LBBB to narrow complex after the patient coughed as shown in Figure 2A-2B.

**Figure 3:**
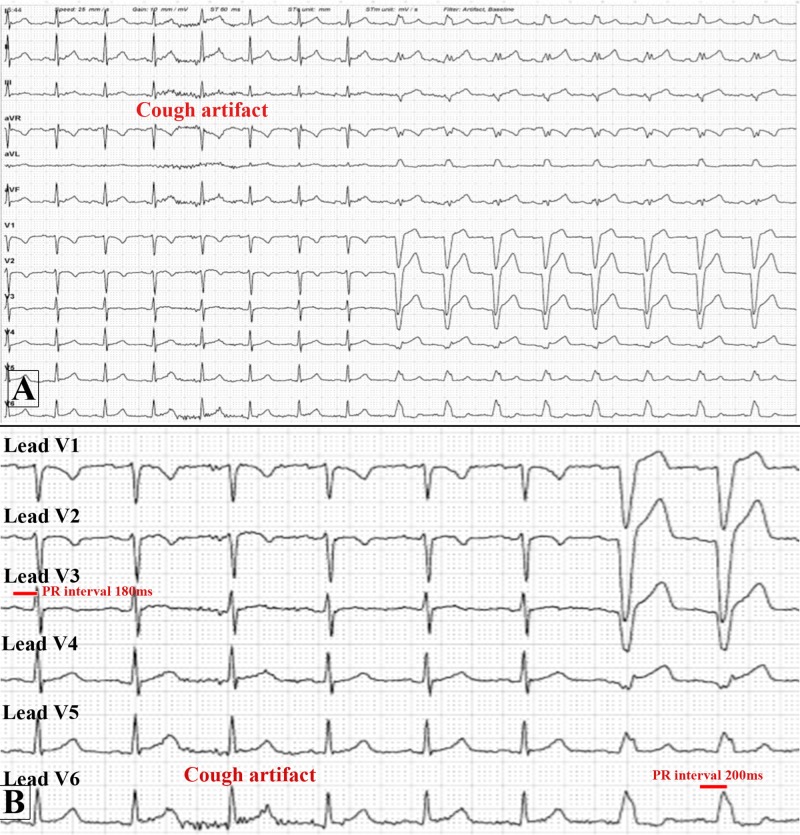
Electrocardiogram (ECG) at the start of the stress part of the myocardial perfusion scan before injecting IV dipyradimole. (A) It shows the conversion of the narrow QRS complexes to left bundle branch block after the patient was asked to cough again as depicted by the artifact in the ECG; (B) Zoomed image of the ECG which shows the precordial leads V1 to V6 showing the subtle increase in PR interval from 180 milliseconds before the cough with narrow QRS complexes to 200 milliseconds afterward with wide QRS complexes.

He was then asked to cough again which showed conversion of narrow complex rhythm back to LBBB as shown in Figure 3A-3B.

**Figure 4:**
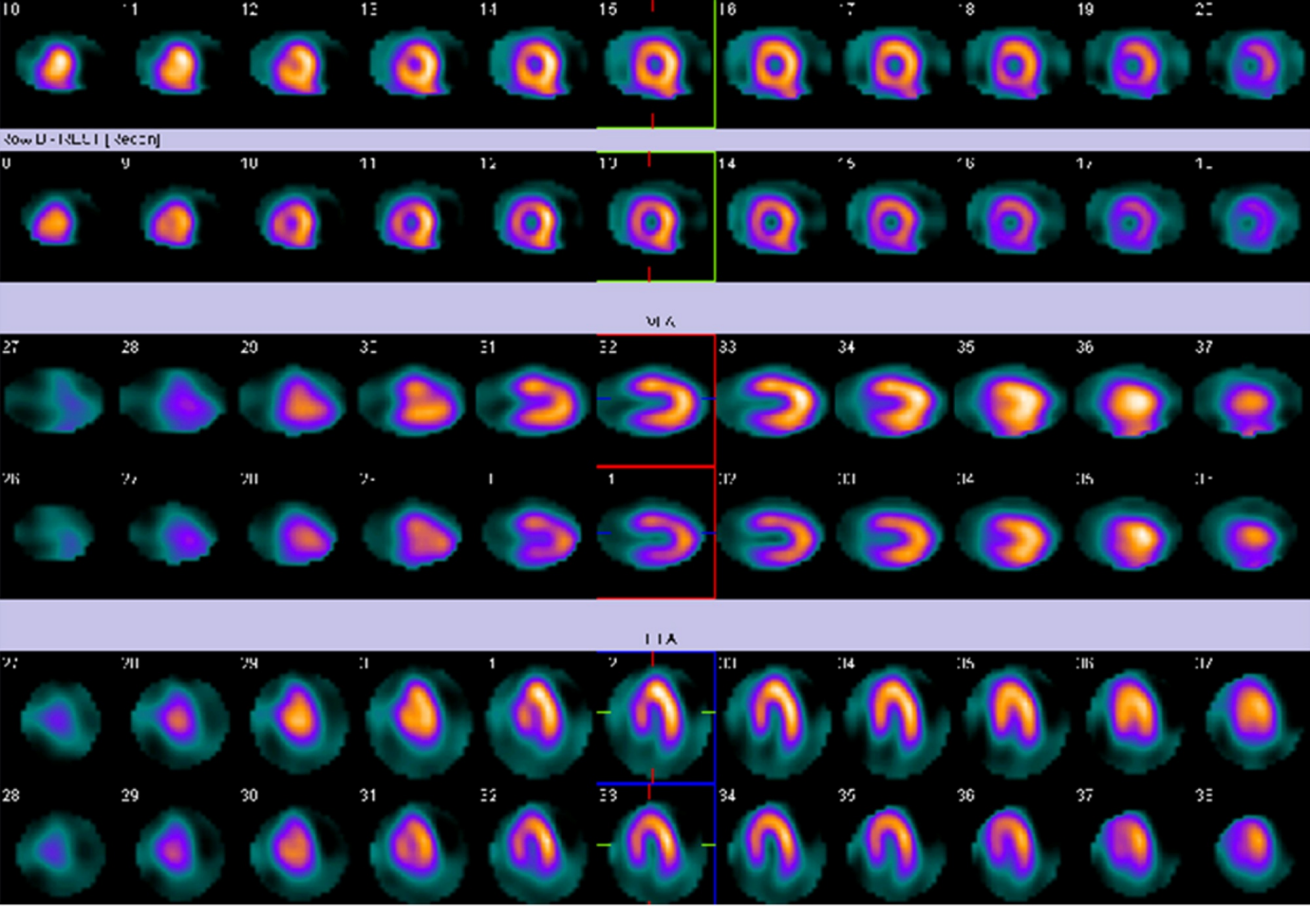
Myocardial perfusion imaging. Myocardial perfusion scan of the patient in warm metal which shows reduced count in the anteroseptal region which is likely attributed to the left bundle branch block on the electrocardiogram.

Then 0.56 mg/kg of IV dipyradimole was injected followed by IV 270 MBq of Tc-99m tetrofosmin for stress SPECT imaging. IV aminophylline was given as per the protocol of myocardial perfusion imaging followed at our center. Neither the IV medications nor the injection of the isotope changed the LBBB to narrow complex rhythm. The patient was then sent for myocardial perfusion imaging. The myocardial perfusion scan was found to have a slightly reduced count in the anteroseptal region likely due to the LBBB as shown in Figure 4. The gated images revealed a calculated ejection fraction of 60% in stress with anteroseptal hypokinesis (not shown).

He was started on oral antacids and oral proton pump inhibitors. It was decided to follow the patient for any recurrent symptoms or development of high-risk features which would necessitate a coronary angiogram. On two months follow-up, the patient remains asymptomatic and can easily walk more than four flights of stairs with no complaints. 

## Discussion

An inter-ventricular conduction defect that returns to normal, whether temporary or otherwise, is termed as a transient bundle branch block; whereas the presence of intermittent conduction defect in a single electrocardiogram alternating with normal, or pre-excitation complexes is termed as an intermittent bundle branch block [6-7].

In 1931, three cases of bundle branch block transitioning to normal conduction were described in response to respiratory maneuvers, partially attributed to vagal effect [8]. A case series published in 1964 reported five cases out of 167 patients with bundle branch block which was intermittent and could be induced with simple maneuvers. Four out of those five patients had underlying ischemic heart disease [9].

Another case of LBBB reported in 2012 was of an elderly man with laughter-induced LBBB, who was found to have a partially reversible inferolateral defect with inducible peri-infarct ischemia. He subsequently had triple vessel disease on coronary angiogram. Successful percutaneous intervention to the left circumflex artery was done after which the LBBB was not induced by laughing, raising the likelihood that the mechanism of LBBB was ischemia-driven [10]. To the best of our knowledge, there is no prior documented case of control of LBBB with coughing.

In our case, the patient had a perfusion defect that is most likely attributed to the presence of the LBBB rather than ischemia. We believe that coughing changes the vagal tone as demonstrated by a change in the PR interval on the ECG which contributes towards the intermittent appearance and disappearance of LBBB similar to the mechanism reported by Herrmann and Ashman [8]. As majority of the cases on voluntary induction of LBBB in our literature review had underlying ischemic heart disease, we decided to follow-up the patient for any repeated symptoms or development of any high-risk features warranting a coronary angiogram. Till date, the patient remains asymptomatic on proton pump inhibitors. 

## Conclusions

Most patients with LBBB are those with prior hypertension, coronary artery disease, or cardiac dilatation. However, our case adds to the handful of cases of voluntary induction of LBBB in literature. It is possible that coughing alters the vagal tone of the body, leading to the conversion of LBBB to narrow complex rhythm and vice versa. Literature review also reveals that mostly voluntary control of LBBB is associated with underlying ischemia. This warrants a follow-up on such patients for the development of typical symptoms or high-risk features.
